# The Effect of Personality on Chrononutrition during the COVID-19 Lockdown in Qatar

**DOI:** 10.3390/nu14132725

**Published:** 2022-06-30

**Authors:** Tamara Al-Abdi, Alexandros Heraclides, Alexia Papageorgiou, Elena Philippou

**Affiliations:** 1 Department of Human Nutrition, Qatar University, Doha P.O. Box 2713, Qatar; 2 Department of Health Sciences, School of Sciences, European University Cyprus, Nicosia 2404, Cyprus; a.heraclides@euc.ac.cy; 3 Department of Primary Care and Population Health, University of Nicosia, Nicosia 1700, Cyprus; papageorgiou.a@unic.ac.cy; 4 Department of Life and Health Sciences, School of Sciences and Engineering, University of Nicosia, Nicosia 1700, Cyprus; philippou.e@unic.ac.cy; 5 Department of Nutritional Sciences, King’s College London, London WC2R 2LS, UK

**Keywords:** COVID-19, lockdown, personality, circadian dysregulation, time-restricted feeding, dietary habits

## Abstract

The COVID-19 lockdown has had a significant impact on people’s lives worldwide. This study aimed to investigate the effect of personality on chrononutrition during the COVID-19 lockdown. Using a cross-sectional design, a convenient sample of 543 adults in Qatar completed an online questionnaire using validated tools to assess personality and chrononutrition behaviors during the first COVID-19 lockdown. Participants scoring high in openness were more likely to eat at night (mean difference (MD) = 0.41, 95% confidence interval (CI): 0.10, 0.72) compared to those scoring high in agreeableness, while those scoring high in extraversion and openness had a shorter eating window (MD = −76.6, 95%CI: −146.3, −6.93 and MD = −29.8, 95%CI: −56.5, −3.01, respectively). Participants high in extraversion had longer evening latency (MD = 66.3, 95%CI: 25.4, 107.3) and evening eating (MD = −62.0, 95%CI: −114.0, −9.0) compared those high in agreeableness. Participants high in conscientiousness showed evidence of first eating event misalignment during the weekend (MD = 22.0, 95%CI: 0.15, 43.9) and last eating event misalignment during weekdays (MD = −27.8, 95%CI: −47.3, −8.41) compared to those high in agreeableness. Lastly, participants high in openness showed evidence of eating window misalignment during the weekend (MD = 30.6, 95%CI: 5.01, 56.2). This study suggests that personality traits can inform personalized nutritional approaches when aiming for healthy habits during unexpected periods, such as the COVID-19 pandemic.

## 1. Introduction

In March 2020, the WHO declared the outbreak of COVID-19 as a pandemic. Various countries all around the world, including Qatar, adopted protective lockdown measures to limit the spread of infection by closing shops, gyms, schools, and universities. Individuals differed in their adherence to these measures, but the origins of these differences are yet to be understood. An emerging body of research suggests that personality traits capture the individual differences observed in health behaviors during the COVID-19 pandemic [1,2,3]. The five-factor model (FFM), a well-known and validated model [4], defines personality with five broad traits: openness, extraversion, agreeableness, neuroticism, and conscientiousness [5]. In relation to the FFM, some studies have reported that individuals scoring high in openness, agreeableness, and conscientiousness and low on neuroticism were significantly more likely to engage in behaviors such as social distancing and taking precautionary measures during the lockdown in the US and Qatar [3,6,7,8]. Other research has shown that, compared to other personality traits, those scoring high in extraversion were more likely to make healthier eating choices during lockdown [1,2]. Those scoring high in conscientiousness were more likely to increase their physical activity [9] compared to those scoring high in neuroticism. Furthermore, high scores in agreeableness and extraversion were associated with positive changes in physical activity [10,11].

One of the main consequences of lockdown measures, which included home confinement and working remotely, is the disruption in chrono-biological rhythms [12,13]. These rhythms are well-defined biological fluctuations in physiological, homeostatic, behavioral, and endocrine functions of an individual within a 24 h period [14]. In the last few years, there has been an increasing interest on the impact of the circadian rhythm on nutrition [15,16,17,18]. This concept, known as ‘chrononutrition’, focusing not just on what we eat but also when we eat, has been associated with many metabolic and hormonal effects [19]. It covers three aspects: (i) (ir)regularity of food intake (having similar amounts of energy intake at mealtimes, from one day to the next); (ii) frequency of food intake (number of meals per day); and (iii) timing of food intake (actual time of day) [19,20]. Potential disrupters of the circadian body rhythm include shift work, social jet lag (delay in the body’s natural sleep clock), sleep restriction, exposure to light at night, and misaligned feeding, all of which have been associated with metabolic alterations [21,22]. Current theories on the topic suggest an association between meal patterns/timings and body weight, which may be caused by a physiological adaptation to sleeping and eating at abnormal circadian times [19,23,24]. It is suggested that consuming irregular meals may affect the circadian body rhythm, including many physiological and metabolic-related processes, such as glycolysis, glycogenesis, and lipid metabolism [19,20,25]. Furthermore, circadian misalignment has been shown to cause changes in circulating satiety hormones, such as leptin and ghrelin, with subsequent effects on energy intake and expenditure [23]. In addition, this misalignment modifies the skeletal muscle clocks, which are the largest collection of peripheral clocks in the body, further leading to irregular energy metabolism and insulin resistance [24]. Studies have also reported that a delay in mealtimes, particularly of dinner, may cause circadian misalignment, due to feeding becoming more at risk of shifting towards the sleeping or resting phase [25].

Internal misalignment has been linked to obesity, diabetes, and cardiovascular diseases [26,27,28]. Studies on shift workers have reported that the energy intake over a 24 h period did not vary compared to that of fixed day workers [29]. However, it is the consumption of meals at the wrong time of the 24 h cycle that was found to be a key contributor to the increased risk of metabolic disturbances [30,31]. Late night feeding and breakfast skipping have also been associated with an increased risk of obesity [15,32]. Another relevant eating pattern is an individual’s eating window, with some studies reporting that time-restricted eating (i.e., a decrease from ≥14 h to 8–10 h eating window) leads to improvements in weight loss, blood pressure, glycemic control, and cholesterol concentration [33,34].

Chrononutrition may be associated with personality traits. Some personality traits were shown to be strongly correlated with eating styles and personal food choices [35]. Those scoring high in conscientiousness, for example, tend to consume meals at regular times of each day and are more organized and self-disciplined compared to those with other personality traits [35]. No known study to date, however, has addressed personality in relation to circadian eating misalignment. Interventions that control the number of meals and/or when the last meal is consumed may potentially enhance the effectiveness of standard weight management programs and improve cardio metabolic health. There are some limited studies that have addressed other facets of circadian rhythm, such as diurnal preferences known as morningness/eveningness (M/E) and chronotype, the behavior associated with sleeping habits [36,37]. They have reported that poor eating habits are related to the evening chronotype, while morningness is positively associated with restrained and healthier eating patterns [37]. Limited studies have found personality traits to be associated with circadian preference. Morning type preferences, and those positively associated with restrained eating, have been linked to high scores of conscientiousness [38,39,40,41] and, to some extent, agreeableness [39,42]. On the other hand, evening-type preferences and preferences positively associated with uncontrolled eating behavior and eating later in the day have been linked to high scores of neuroticism [43,44,45]. The remaining personality traits (extraversion and openness) are less consistent [43] and might even be unrelated to time of day preference [46]. Most of these studies, however, used Eysenck’s three factor personality model to assess personality characteristics, based only on three dimensions, namely extraversion, neuroticism, and psychoticism [47]. The FFM, however, is one of the best known and most frequently used validated instruments in studies investigating the correlation between personality and health outcomes, mainly due to its clear factor structure, reliability, and validity when compared with other measures of personality [4,48]. Furthermore, although some studies have reported the effects of the COVID-19 lockdown on circadian rhythm and sleep [49,50], none have addressed chrononutrition and whether this is associated with personality. The aim of this study is thus to investigate, for the first time, the association between personality and chrononutrition during the COVID-19 lockdown. This is especially relevant in Qatar, where no research has been done in this area.

## 2. Materials and Methods

### 2.1. Study Design and Participants

A cross-sectional study was conducted from 24 April to 23 May 2020 in Qatar, during which time Qatar was under COVID-19 lockdown, which included social distancing, home confinement, and the closure of businesses and schools. The Qatari population were required to stay at home and only go out for essential needs (food or medicine). For those who did leave the house, only one individual was allowed to be in the car. With regards to work, only essential workers, such as health care workers, were allowed out with physical distancing guidelines. The target population was residents of Qatar, males or females, aged 18 years or older recruited through social media (WhatsApp and Twitter) and by email using a snowball convenience sampling method. Respondents were asked to complete an online questionnaire anonymously that was distributed through social media and encouraged to distribute the questionnaire to their contacts residing in Qatar. Despite the initial sampling approach, it became apparent that snowball sampling was not followed by the majority of participants, rendering our sample more like a “convenient” sample rather than a “snowball” sample. Participants were asked to provide written informed consent prior to completing the questionnaire. Ethical approval for this study was obtained from Qatar University Institutional Review Board (QU-IRB 1081-EA/19), and the study was conducted in accordance with the Declaration of Helsinki.

### 2.2. Questionnaire

The questionnaire consisted of questions structured into three sections: (1) socio demographics, (2) personality, and (3) chrononutrition, as described in detail below.

#### 2.2.1. Socio-Demographic Data

Participants were asked to report their age (≤20 years; 21–29 years; 30–39 years; ≥40 years), gender (male or female), nationality (Arab or non-Arab), highest education attained (secondary school; university undergraduate; university postgraduate), marital status (single, married), work status (student, employed, unemployed), smoking status (yes or no), and weight and height during the lockdown. Self-reported data on height and weight were used to calculate BMI (kg/m^2^) and interpreted according to the WHO classification as underweight (BMI: <18.5 kg/m^2^), normal weight (BMI: 18.5–24.9 kg/m^2^), overweight (BMI: 25.0–29.9 kg/m^2^), and obese (BMI: ≥30 kg/m^2^).

#### 2.2.2. Personality

Personality traits were assessed using the Big Five Inventory–BFI-44, a 44-item validated short-version questionnaire that classifies an individual into five broad dimensions: agreeableness, extraversion, conscientiousness, neuroticism, and openness based on the FFM [51]. The BFI-44 is one of the most commonly used instruments in studies about personality, given its reliability and validity in diverse and cross-cultural samples [52]. Participants were asked to report their level of agreement with statements about how they see themselves on a five-point scale ranging from 1 = strongly disagree to 5 = strongly agree. An eight-item scale was used to assess agreeableness (e.g., “I see myself as someone who is helpful and unselfish with others”). A nine-item scale was used to assess extraversion (e.g., “… is outgoing, sociable”). A nine-item scale was used to assess conscientiousness (e.g., “… does a thorough job”). An eight-item scale was used to assess neuroticism (e.g., “… gets nervous easily”). A ten-item scale was used to assess openness (e.g., “… is curious about many different things”). Items were reverse-scored where necessary, and the mean was taken across items for each trait, with higher scores indicating a higher observance of the traits.

#### 2.2.3. Chrononutrition

The Chrononutrition Profile-Questionnaire (CP-Q) was used to assess chrononutrition behaviors, preferences, and eating misalignment [53]. Although it is a fairly new tool, CP-Q is a concise and thorough measurement of chrononutrition [53]. It is easily administered with little instruction, and its validity and reliability have been tested [53,54]. The questionnaire consists of 18 items (open-ended and multiple-choice options) designed to assess both chrononutrition behaviors and preferences during an average week (on typical work/school and free/weekday days). Six items were used to assess chrononutrition behaviors: (i) breakfast skipping: number of days per week with no breakfast consumption; (ii) largest meal: meal in which the largest amount of calories is consumed; (iii) night eating: number of days per week eating after initial sleep onset; (iv) eating window: duration of time between the first and last eating event of the day in minutes; (v) evening latency: duration of one’s last eating event and sleep onset in minutes; (vi) evening eating: eating late in the waking day in hours and minutes (HH:MM). Five items were used to assess chrononutrition preferences: preferred first and last eating events, preferred morning and evening latency, and preferred eating window (“if you were entirely free to plan your day….”). Scores were calculated following the CP-Q scoring protocol. In brief, continuous values were extracted for each chrononutrition behavior, and a weighted aggregate score was calculated to represent weekly patterns (both workdays and free days).

Eating misalignment, the discrepancy between preferred and actual chrononutrition behaviors, was calculated by subtracting each participant’s reported chrononutrition behavior from their reported chrononutrition preference, for the following variables of interest: first eating event, morning latency, last eating event, evening latency, and eating window. Thus, if an individual was eating earlier than preferred, this would be represented as a positive value, whereas if an individual was eating later than preferred, this would be represented as a negative value. Eating alignment was calculated by subtracting the participant’s reported chrononutrition behavior from the preference for first eating event, morning latency, last eating event, evening latency, and eating window. Eating alignment was defined as eating within 30 min of one’s preferred first and last eating event times and morning and evening latencies, or within 60 min of one’s preferred eating window. Values beyond these cutoffs were considered eating misalignment. Permission to use the CP-Q was obtained by email communication with Dr Allison Veronda (who developed and validated the questionnaire) prior to carrying out the study.

### 2.3. Statistical Analysis

Descriptive statistics are presented as number and percentage (%) for categorical variables and mean ± standard deviation for continuous variables. Distribution of numeric variables was assessed using normality plots. Baseline characteristics of participants were evaluated by personality type using the chi-squared test. Associations of personality dimensions with chrononutrition variables were investigated using multiple linear regression models using personality as the main independent variable of interest and numeric chrononutrition outcomes, in turn, as dependent variables. No adjustments were made in the first regression model. The second model was adjusted for gender, age, nationality, education, marital status, employment status, smoking, and BMI since these factors are associated with both personality and chrononutrition and could thus act as potential confounders. Estimates from linear regression analyses represent mean difference (95% confidence intervals, 95%CI) in the outcome variable (chrononutrition measure) comparing all personality categories to a reference category (agreeableness). All linear regression assumptions (linearity, normality of residuals, homoscedasticity) were met in all of the regression models. It should be noted that no specific methods for dealing with “nested” data (e.g., cluster-robust standard errors) were required, since the extent of snowball sampling was very limited (see Section 2.1).

Similarly, categorical chrononutrition outcomes were investigated using multiple logistic regression models, following the same adjustment process described above. Estimates from logistic regression analyses represent odds ratios (95%CI) for the likelihood of the outcome variable (chrononutrition measure) comparing all personality categories to the reference category (agreeableness). Variables in time format (HH:MM) were converted to minutes for regression analyses. Results were considered statistically significant at the 5% level (*p*-value < 0.05). Data analysis was done using STATA (Version 16, Stata Corporation, College Station, TX, USA).

## 3. Results

### 3.1. Participants’ Sociodemographic Characteristics

Six hundred and five online questionnaires were sent out, of which a total of 560 (92.5% response rate) responses were received. Seventeen questionnaires were excluded due to incomplete data, leaving a final sample of 543 participants. The demographic characteristics of the participants are shown in Table 1. In brief, most of the participants were female (88.4%), and almost half (44.8%) were aged between 21 and 29 years. Most were Arab (73.7%), students (46%), had an undergraduate level of education (77.7%), and were single (63.4%) and nonsmokers (91.9%). Regarding body weight, 31.3% were classified as overweight and 13.3% were classified as obese. With respect to personality, the majority of participants scored high in openness (41.4%), while females were overrepresented among those scoring high in openness (42.7%), and males were overrepresented among those scoring high in conscientiousness (47.6%) (*p* ˂ 0.001). With regards to body weight, those with high scores in agreeableness were more likely to be under or normal weight, while those with high scores in openness were more likely to be overweight and obese (*p* ˂ 0.001).

### 3.2. Personality and Chrononutrition during the COVID-19 Lockdown

Descriptive statistics of chrononutrition behaviors are presented in Table 2. Eating window and evening latency variables are reported in minutes, and evening eating in time format (HH:MM). For regression analysis, time format was converted to minutes. Associations between personality and weekly averaged chrononutrition behaviors as assessed by the CP-Q (breakfast skipping, night eating, eating window, evening latency, evening eating, and largest meal consumed) were found in this study. Results after adjusting for confounders are presented in Table 3. Individuals who scored high in openness were more likely to engage in night eating compared to those who scored high in agreeableness (*p* = 0.010). Individuals who scored high in extraversion and openness had a shorter eating window than those who scored high in agreeableness, by 76.6 and 29.8 min, respectively (*p* = 0.031, *p* = 0.029). For those with high scores of extraversion, a longer evening latency was observed by 66.6 min compared to those with high scores in agreeableness (*p* = 0.002). Those scoring high in extraversion were also more likely to engage in evening eating compared to those who scored high in agreeableness (*p* = 0.020). No associations were found between personality traits and breakfast skipping. Results after adjusting for confounders are presented in Table 4. Results for the association between chrononutrition and personality before adjusting for cofounders, not discussed here, can be found in Supplementary Tables S1 and S2.

### 3.3. Personality and Eating Misalignment during the COVID-19 Lockdown

With regards to eating alignment (eating within 30 min of one’s preferred first and last eating event times and morning and evening latencies, or within 60 min of one’s preferred eating window), personalities varied across chrononutrition behaviors. Results after adjusting for confounders are presented in Table 5. The first eating event on the weekend of those who scored high in conscientiousness was 22 min later than those who scored high in agreeableness (112.9 min), with a significant association of *p* = 0.048. This is indicative of eating misalignment, as it exceeded eating within 30 min of waking up. An eating misalignment was also found in those who scored high in conscientiousness for the last eating event on the weekday, which was 28 min earlier than for those who scored high in agreeableness (86.1 min), with a significant association of *p* = 0.005. The eating window for participants with high scores of openness was also misaligned on the weekend, being 30.6 min later compared to those who scored high in agreeableness (109.5 min), with a significant association of *p* = 0.019. Results for the association between chrononutrition alignment and personality before adjusting for confounders, not discussed here, can be found in Supplementary Table S3.

## 4. Discussion

This study assessed for the first time the association of personality and chrononutrition behaviors including eating misalignment. Our findings suggest that some personality traits such as openness, extraversion, and conscientiousness might be linked to chrononutrition behaviors during the COVID-19 lockdown. Participants with high scores in openness were more likely to eat at night, while participants with high scores in extraversion and openness were more likely to have a shorter eating window. High scores in extraversion were associated with evening eating and a longer evening latency. In relation to circadian misalignment, high scores in conscientiousness were associated with first eating event misalignment during the weekend and last eating event misalignment during the weekday. Lastly, high scores in openness were associated with eating window misalignment during the weekend. These results may have important implications for understanding how differences in personality traits predict the ways in which individuals’ eating habits can impact their health during unprecedented times, such as those of a pandemic and lockdown.

Our finding that those who scored high in openness were more likely to exhibit night eating was also shown in other studies linking personality traits to chronotype preferences [38,39]. People scoring high in openness tend to engage in social activities that keep them up later at night, and this may affect their consumption of food. Several studies have found that late meal intake is indicative of excessive total daily intake [45,55], weight gain [23], and higher BMI [56,57]. Indeed, recent studies have shown that eating timings may be as important as macronutrient composition in controlling changes in bodyweight [25,26,58]. This might have implications for weight management interventions, since individuals with high scores in openness may be predisposed to engage in energy intake at night due to personality-related eating habits.

High scores in extraversion and openness were associated with a shorter eating window. This might have beneficial implications for certain personality traits, since studies have shown that a longer eating window may contribute to the onset of chronic disease [59]. Daily meal patterns spread over a period longer than 12 h—compared to time-restricted feeding (TRF), in which all nutrient intake occurs within 12 h or less—have been linked to an increase in body weight [60]. Our finding that scoring high in extraversion was associated with evening eating and longer evening latency is of similar importance. This is because higher energy intake during the two hours before bedtime has been associated with a fivefold increased risk in obesity [61]. Furthermore, a recent longitudinal study in the US reported that greater % of energy consumed within the night window (closer to bedtime) has been associated with an 80% chance of being overweight or obese [58].

Interestingly, regarding eating misalignment, our study found that high scores in conscientiousness are associated with misalignment for both the first eating event on weekends and the last eating event on weekdays. The eating window for those scoring high in openness was also found to be misaligned on weekends. Our findings do contradict most research related to chronotype and eating habits, which report consistently positive correlations between those scoring high in conscientiousness and morningness [47]. A possible explanatory reason for this discrepancy could be the circumstances of the COVID-19 lockdown. Indeed, a recent study on the impact of COVID-19 pandemic on different chronotype profiles reported an unexpected increase in the amount of food intake consumed near bedtime for those with a morning chronotype [62]. They found that those with a morningness profile were less likely to maintain regular schedules during the pandemic period. The few studies on the effect of COVID-19 pandemic on personality have yielded mixed results. Although the majority of studies have reported conscientiousness to be associated with positive health behaviors [3], one study by Kohut et al. [63] reported that conscientiousness was not a predictor of any preventive measures related to COVID-19. Similarly, the majority of studies have reported a negative association of neuroticism with an increase in non-beneficial health outcomes and risk behaviors [64]; however, Kekalainen et al. [1] reported that those with high scores in neuroticism were more likely to adhere to social distancing guidelines.

Although FFM personality traits are known to be stable throughout an individual’s life, some studies have reported the pronounced effects of stressful and adverse life events on personality traits [65,66]. The COVID-19 pandemic’s disruption of daily life could possibly have an effect on personality. Indeed, a study by Sutin et al. [67] found a small change in the FFM personality traits during the acute phase of the pandemic in a US population sample of 2317 adults. Using a pre/post-test design to assess whether the five factor model personality traits changed during COVID-19 lockdown, they found that during six weeks of isolation, neuroticism unexpectedly decreased rather than increased. Furthermore, they reported a decline in conscientiousness and openness traits during isolation, suggesting that situational factors related to stress and anxiety play a role. It could be that those who score high in conscientiousness and openness may not adhere to self-imposed goals normally seen with these type of traits. They may have less pressure to be organized or complete tasks in a timely manner, thus impacting meal timing and causing an eating misalignment as shown in our study. Emotional stability, achievement motivation, and self-regulation, normally associated with these personality traits [68], may be impacted during COVID-19 lockdown. The pandemic, with its unprecedented disruption of daily life, could also possibly have an unprecedented effect on personality.

This study has a number of strengths and limitations. With regards to limitations, the study population had a high proportion of participants who were single female students, aged 21–29 years, with an undergraduate degree education level, thus limiting the generalizability of the findings. This was due to the convenience nature of the sampling method, and as recruitment was conducted mostly through social media, it mainly targeted younger people. The information gathered on chrononutrition and personality was self-reported, which could lead to recall bias; nevertheless, this is common practice in research involving these factors. No data was collected on menstrual cycle timing, which could have an impact on eating habits [69,70]. Moreover, the cross-sectional design of the study and the lack of information on the participants’ chrononutriton behavior before the lockdown do not allow us to draw conclusions on cause and effect relationships. The main strength of this study was that it provided, for the first time, observational data on associations between chrononutrition and personality dimensions during the COVID-19 lockdown in Qatar. The online questionnaire was a quick and cost-effective method of gathering data, since a face-to-face questionnaire would have been impossible to administer due to the social distancing measures enforced by the lockdown. Longitudinal studies with representative samples should be conducted to better understand the lasting effects of the COVID-19 pandemic and chrononutrition in association with the different personality dimensions. Chrononutrition behaviors may provide innovative health and weight management interventions, which could be beneficial during the current COVID-19 pandemic.

## 5. Conclusions

The present exploratory study highlights the significant impact of the COVID-19 lockdown in Qatar on the association between personality and chrononutrition. This emphasizes the importance of addressing differences in how individuals respond to the pandemic and understanding how personality can predict what and when people eat when faced with a novel environment. The association between chronotype and personality might help further explain the social influences on circadian preferences and alignment. Adjustments to meal timings are an attractive strategy to synchronize circadian rhythms in relation to personality traits. The role of personality in dietary modification of meal timings should be examined further and could serve as an effective tool for reducing risk of cardio-metabolic diseases and obesity, especially during any potential future outbreaks.

## Figures and Tables

**Table 1:** Characteristics of the studied population by personality during the COVID-19 lockdown.

		Personality Traits						
		All	Extraversion	Agreeableness	Conscientiousness	Neuroticism	Openness	*p*-Value
Variables		(*n* = 543)	(*n* = 19)	(*n* = 179)	(*n* = 105)	(*n* = 15)	(*n* = 225)	
Gender	Female	480 (88.4)	16 (84.2)	171 (95.5)	75 (71.4)	13 (86.7)	205 (91.1)	˂0.001
	Male	63 (11.6)	3 (15.8)	8 (4.5)	30 (28.6)	2 (13.3)	20 (8.9)	
Age (years)	≤20	113 (20.8)	3 (15.8)	51 (28.5)	13 (12.4)	1 (6.7)	45 (20.0)	˂0.001
	21–29	243 (44.8)	10 (52.6)	86 (48.0)	33 (31.4)	8 (53.3)	106 (47.1)	
	30–39	99 (18.2)	2 (10.5)	22 (12.3)	28 (26.7)	2 (13.3)	45 (20.0)	
	≥40	88 (16.2)	4 (21.1)	20 (11.2)	31 (29.5)	4 (26.7)	29 (12.9)	
Nationality	Non-Arab	143 (26.3)	6 (31.6)	41 (22.9)	41 (39.1)	3 (20.0)	52 (23.1)	0.020
	Arab	400 (73.7)	13 (68.4)	138 (77.1)	64 (61.0)	12 (80.0)	173 (76.9)	
Education	Secondary	28 (5.2)	1 (5.2)	10 (5.6)	2 (1.9)	0 (0.0)	15 (6.7)	0.004
	Undergraduate	422 (77.7)	15 (79)	155 (86.6)	77 (73.3)	13 (86.7)	162 (72.0)	
	Postgraduate	93 (17.1)	3 (15.8)	14 (7.8)	26 (24.8)	2 (13.3)	48 (21.3)	
Marital Status	Single	344 (63.4)	13 (86.4)	129 (72.1)	41 (39.0)	7 (46.7)	154 (68.4)	˂0.001
	Married	199 (36.7)	6 (31.6)	50 (27.9)	64 (61.0)	8 (53.3)	71 (31.6)	
Work Status	Student	250 (46.0)	9 (47.4)	108 (60.3)	24 (22.9)	4 (26.7)	105 (46.7)	˂0.001
	Employed	76 (14.0)	3 (15.8)	24 (13.4)	11 (10.5)	4 (26.7)	34 (15.1)	
	Unemployed	217 (40.0)	7 (36.8)	47 (26.3)	70 (66.7)	7 (46.7)	86 (38.2)	
Smoker	No	499 (91.9)	17 (89.5)	174 (97.2)	93 (88.6)	14 (93.3)	201 (89.3)	0.033
	Yes	44 (8.1)	2 (10.5)	5 (2.8)	12 (11.4)	1 (6.7)	24 (10.7)	
BMI	Underweight	44 (8.1)	4 (21.0)	15 (8.4)	8 (7.6)	1 (6.7)	16 (7.1)	˂0.001
	Normal	257 (47.3)	12 (63.2)	111 (62.0)	39 (37.2)	8 (53.3)	87 (38.7)	
	Overweight	170 (31.3)	3 (15.8)	39 (21.8)	50 (47.6)	6 (40.0)	72 (32.0)	
	Obese	72 (13.3)	0 (0.0)	14 (7.8)	8 (7.6)	0 (0.0)	50 (22.2)	

Statistical significance at the 5% significance level using chi-squared test. *n* = frequency; % = percentage.

**Table 2:** Descriptive statistics of chrononutrition behaviors during the COVID-19 Lockdown.

Chrononutrition Behaviors			
Variables	Format	Mean (SD)	Range
Breakfast skipping	Days	2.20 (2.40)	0.00–7.00
Night eating	Days	0.50 (1.30)	0.00–7.00
Eating window aggregate	Mins	727.3 (129.5)	240.0–1114.0
Evening latency aggregate	Mins	177.9 (82.2)	0.0–477.0
Evening eating aggregate	HH:MM	20:36 (3:15)	18:00–2:17
Largest Meal	*n* (%)		
Breakfast	31 (5.7%)		
Lunch	420 (47.3%)		
Dinner	92 (17%)		

Eating window, evening latency, and evening eating variables are presented as a calculated aggregate score, weighed to represent five workdays and two weekend days.

**Table 3:** Mean differences (95% confidence intervals) for the association between numeric chrononutrition outcomes and personality traits during the COVID-19 lockdown, assessed by linear regression.

		Personality Traits			
Chrononutrition Variables	Agreeableness	Extraversion	Conscientiousness	Neuroticism	Openness
Breakfast Skipping (Days)	Ref (1.40) ^a^	−0.29 (−1.48, 0.89)	−0.01 (−0.65, 0.63)	−0.70 (−1.78, 0.37)	0.38 (−0.13, 0.89)
Night Eating (Days)	Ref (0.33) ^a^	−0.23 (−0.58, 0.13)	−0.15 (−0.41, 0.11)	−0.18 (−0.58, 0.21)	0.41 * (0.10, 0.72)
Eating Window (Mins)	Ref (776.6) ^a^	−76.6 * (−146.3, −6.93)	−14.1 (−47.5, 19.4)	12.3 (−44.9, 69.5)	−29.8 * (−56.5, −3.01)
Evening Latency (Mins)	Ref (138.0) ^a^	66.3 * (25.4, 107.3)	−5.29 (−25.9, 15.4)	−16.1 (−60.1, 27.8)	−6.69 (−24.0, 10.7)
Evening Eating (Mins)	Ref (588.0) ^a^	−62.0 * (−114.0, −9.0)	−33.0 (−77.0, 10.0)	18.0 (−28.0, 65.0)	−35.0 (−81.0, 10.0)

Linear regression model adjusted for gender, age, nationality, education, marital status, work status, BMI, and smoking with numeric chrononutrition as dependent variables and personality traits as the independent variable; mean difference (95% CI) between each personality trait and the personality trait agreeableness (reference category). * Values indicate statistical significance at the 5% significance level using linear regression. ^a^ indicates mean of reference category (agreeableness). Breakfast skipping: days per week no breakfast consumption. Night eating: days per week eating after initial sleep onset. Eating window: duration of time between first eating event and last eating event of the day in minutes. Evening latency: duration of one’s last eating event and sleep onset in minutes. Evening eating: eating late in waking day in minutes. Eating window, evening latency, and evening eating variables are presented as a calculated aggregate score, weighed to represent five workdays and two weekend days.

**Table 4:** Odds ratios (95% confidence intervals) for the association between binary chrononutrition outcomes and personality traits during the COVID-19 lockdown, assessed by logistic regression.

		Personality Traits			
Chrononutrition Variables	Agreeableness	Extraversion	Conscientiousness	Neuroticism	Openness
Largest meal (Lunch)	Ref ^a^	0.49 (0.16, 1.52)	0.66 (0.36, 1.22)	2.28 (0.37, 14.2)	1.29 (0.74, 2.25)

Logistic regression model adjusted for gender, age, nationality, education, marital status, work status, BMI, and smoking. Values indicate statistical significance at the 5% significance level using logistic regression. Logistic regression using a dummy variable was used for comparison of the largest meal variable and personality, results are presented as odd ratios (95% CI). ^a^ indicates mean of reference category (agreeableness).

**Table 5:** Mean differences (95% confidence intervals) for the association between chrononutrition alignment and personality during the COVID-19 lockdown.

		Personality Traits			
Chrononutrition Alignment Variables	Agreeableness	Extraversion	Conscientiousness	Neuroticism	Openness
First Eating Event Weekday (Mins)	Ref (153.8) ^a^	−35.6 (−72.6, 1.30)	−18.2 (−49.4, 13.0)	0.61 (−48.6, 49.8)	−16.8 (−43.9, 10.3)
Weekend (Mins)	Ref (112.9) ^a^	−10.1 (−48.3, 28.1)	22.0 * (0.15, 43.9)	−12.0 (−55.0, 31.0)	10.0 (−7.55, 27.6)
Last Eating Event Weekday (Mins)	Ref (86.1) ^a^	10.3 (−33.0, 53.6)	−27.8 * (−47.3, −8.41)	0.10 (−36.6, 36.7)	0.73 (−15.9, 17.4)
Weekend (Mins)	Ref (80.1) ^a^	−13.6 (−46.3, 19.1)	−13.2 (−36.2, 9.80)	28.5 (−6.64, 63.6)	9.58 (−9.33, 28.5)
Morning Latency Weekday (Mins)	Ref (14.3) ^a^	15.5 (−27.2, 58.3)	14.4 (−4.81, 33.6)	3.72 (−28.6, 36.0)	6.25 (−10.4, 22.9)
Weekend (Mins)	Ref (29.8) ^a^	8.18 (−21.5, 37.9)	10.3 (−5.12, 25.8)	19.1 (−12.0, 50.2)	2.39 (−8.32, 13.1)
Evening Latency Weekday (Mins)	Ref (37.8) ^a^	21.0 (−26.8, 68.8)	1.49 (−18.0, 21.0)	25.9 (−19.2, 71.0)	12.4 (−2.90, 27.7)
Weekend (Mins)	Ref (54.0) ^a^	22.1 (−26.9, 71.0)	−3.34 (−22.2, 15.5)	20.4 (−19.6, 60.4)	10.4 (−5.92, 26.6)
Eating Window Weekday (Mins)	Ref (147.9) ^a^	−9.41 (−61.6, 42.8)	−12.1 (−41.5, 17.3)	17.3 (−42.3, 76.8)	7.94 (−20.1, 36.0)
Weekend (Mins)	Ref (109.5) ^a^	24.6 (−20.0, 69.2)	13.7 (−12.5, 39.9)	7.80 (−29.0, 44.6)	30.6 * (5.01, 56.2)

Linear regression model adjusted for gender, age, nationality, education, marital status, work status, BMI, and smoking. Mean difference (95%CI) between each personality trait and the personality trait agreeableness (reference category), presented as minutes. * values indicate statistical significance at the 5% significance level using linear regression. ^a^ indicates mean of reference category (agreeableness). First eating event (time of first meal of the day). Last eating event (time of last meal of the day). Morning latency (duration of time between waketime and first eating event). Evening latency (duration of time between last eating event and sleep onset time). Eating window (duration of time between first and last eating event of the day).

## Data Availability

The data presented in this study are available on request from the corresponding author.

## Supplementary Materials

The following supporting information can be downloaded at: https://www.mdpi.com/article/10.3390/nu14132725/s1, Table S1: Mean Differences (95% Confidence Intervals) for the association between numeric chrononutrition outcomes and personality traits during the COVID-19 lockdown, assessed by linear regression; Table S2: Odds Ratios (95% Confidence Intervals) for the association between binary chrononutrition outcomes and personality traits during the COVID-19 Lockdown, assessed by logistic regression; Table S3: Mean differences (95% Confidence Intervals) for the association between chrononutrition alignment and personality during the COVID-19 Lockdown.
