# Supplementary material for: The Effect of Personality on Chrononutrition during the COVID-19 Lockdown in Qatar
Source: Nutrients. 2022 Jun 30;14(13):2725. doi: 10.3390/nu14132725 (PMC9268339; doi:10.3390/nu14132725)
Supplement: Supplementary file 1 [file nutrients-14-02725-s001.zip › nutrients-1710688-supplementary.pdf]

nutrients

MDPI

*Supplementary Materials*

# The Effect of Personality on Chrononutrition during the COVID-19 Lockdown in Qatar

**Tamara Al-Abdi [1,*], Alexandros Heraclides [2], Alexia Papageorgiou [3] and Elena Philippou [4,5]**

**Table S1.** Mean Differences (95% Confidence Intervals) for the association between numeric chrononutrition outcomes and personality traits during the COVID-19 lockdown, assessed by linear regression.

| | Personality Traits | | | | |
|---|---|---|---|---|---|
| **Chrononutrition Variables** | **Agreeableness** | **Extraversion** | **Conscientiousness** | **Neuroticism** | **Openness** |
| Breakfast Skipping (Days) | Ref (2.11)[a] | −0.29 (−1.40, 0.82) | −0.00 (−0.63, 0.63) | −0.73 (−1.77, 0.31) | 0.41 (−0.09, 0.91) |
| Night Eating (Days) | Ref (0.43)[a] | −0.26 (−0.57, 0.05) | −0.27 * (−0.49, −0.05) | −0.28 (−0.63, 0.07) | 0.35 * (0.05, 0.65) |
| Eating Window (Mins) | Ref (749.3)[a] | −85.5 * (−154.0, −16.9) | −24.5 (−57.4, 8.42) | 7.95 (−49.1, 65.0) | −34.8 * (−60.8, −8.73) |
| Evening Latency (Mins) | Ref (175.1)[a] | 66.6 * (28.7, 104.4) | −5.50 (−14.3, 25.3) | −13.7 (−56.1, 28.7) | −0.87 (−18.0, 16.3) |
| Evening Eating (Mins) | Ref (540.0)[a] | −60.0 * (−111.0, − 10.0) | −42.0 * (−79.0, −4.0) | 14.0 (33.0, 61.0) | −37.0 (−79.0, 5.0) |

Breakfast skipping: # of days per week no breakfast consumption. Night eating: # of days per week eating after initial sleep onset. Eating window: duration of time between first eating event and last eating event of the day in minutes. Evening latency: du-ration of one's last eating event and sleep onset in minutes. Evening eating: eating late in waking day in minutes. Eating window, evening latency and evening eating variables are presented as a calculated aggregate score, weighed to represent five workdays and two weekend days.
Linear regression unadjusted model with numeric chrononutrition as dependent variables and personality traits as the independent variable; Mean difference (95% CI) between each personality trait and the personality trait agreeableness (reference category).
* Values indicate statistical significance at the 5% significance level using linear regression. a indicates mean of reference category (agreeableness).

**Table S2.** Odds Ratios (95% Confidence Intervals) for the association between binary chrononutrition outcomes and personality traits during the COVID-19 Lockdown, assessed by logistic regression.

| | Personality Traits | | | | |
|---|---|---|---|---|---|
| **Chrononutrition Variables** | **Agreeableness** | **Extraversion** | **Conscientiousness** | **Neuroticism** | **Openness** |
| Largest Meal (Lunch) | Ref | 0.37 (0.13, 1.06) | 0.49 * (0.28, 0.87) | 1.44 (0.30, 6.83) | 1.05 (0.63, 1.77) |

Logistic regression unadjusted model.
* Values indicate statistical significance at the 5% significance level using logistic regression. Logistic regression using a dummy variable was used for comparison of the largest meal variable and personality, results are presented as Odd Ratios (95% CI).
[a] indicates mean of reference category (agreeableness).

**Table S3.** Mean differences (95% Confidence Intervals) for the association between chrononutrition alignment and personality during the COVID-19 Lockdown.

| | Personality Traits | | | | |
|---|---|---|---|---|---|
| **Chrononutrition Alignment Variables** | **Agreeableness** | **Extraversion** | **Conscientiousness** | **Neuroticism** | **Openness** |

| | | | | | |
|---|---|---|---|---|---|
| First Eating Event Weekday (Mins) | Ref (153.3)[a] | −49.2 * (−87.3, −11.0) | −32.4 * (−61.6, −3.19) | −17.1 (−60.3, 26.0) | −21.8 (−48.4, 4.89) |
| Weekend (Mins) | Ref (90.8)[a] | −14.7 (−52.0, 22.7) | 9.20 (−12.3, 30.7) | −22.8 (−68.5, 22.9) | 3.65 (−14.1, 21.3) |
| Last Eating Event Weekday (Mins) | Ref (94.2)[a] | 9.97 (−32.8, 52.8) | 35.3 * (−54.3, −16.3) | −8.8 (−42.6, 25.1) | −1.49 (−18.3, 15.3) |
| Weekend (Mins) | Ref (93.2)[a] | −19.1 (−54.0, 15.8) | −21.3 (−43.2, 0.65) | 15.3 (−11.5, 42.0) | 7.41 (−11.6, 26.4) |
| Morning Latency Weekday (Mins) | Ref (63.6)[a] | 15.9 (−27.7, 59.4) | 14.6 (−4.31, 33.4) | 3.37 (− 29.5, 36.3) | 6.05 (− 10.1, 22.2) |
| Weekend (Mins) | Ref (42.8)[a] | 7.53 (−22.2, 37.2) | 12.5 (−2.03, 27.1) | 16.1 (−16.8, 48.9) | 2.23 (−8.68, 13.1) |
| Evening Latency Weekday (Mins) | Ref (73.2)[a] | 18.5 (−27.4, 64.4) | −0.71 (−19.5, 18.0) | 21.4 (−23.2, 66.0) | 13.0 (−1.96, 27.9) |
| Weekend (Mins) | Ref (73.9)[a] | 21.4 (−25.9, 68.7) | − 4.05 (−21.5, 13.4) | 18.4 (−20.2, 57.0) | 9.68 (−5.94, 25.3) |
| Eating Window Weekday (Mins) | Ref (151.0)[a] | − 21.7 (−75.5, 32.1) | − 19.9 (−48.4, 8.54) | 9.49 (−49.7, 68.7) | 3.63 (−23.9, 31.2) |
| Weekend (Mins) | Ref (113.3)[a] | 19.9 (−25.7, 65.4) | 2.65 (−21.9, 27.2) | − 5.01 (−38.8, 28.8) | 26.0 * (1.81, 50.3) |

Unadjusted linear regression model.

Mean difference (95% CI) between each personality trait and the personality trait agreeableness (reference category), presented as minutes.

* values indicate statistical significance at the 5% significance level using linear regression. a indicates mean of reference category (agreeableness). First eating event (time of first meal of the day). Last eating event (time of last meal of the day). Morning latency (duration of time between waketime and first eating event). Evening Latency (duration of time between last eating event and sleep onset time). Eating window (duration of time between first and last eating event of the day).
